# Mechanism and Treatment of Placenta Previa1Read before the Alumni Association of the Washington University Medical School, February 25, 1909. From the University Bulletin.

**Published:** 1909-08

**Authors:** Henry Schwarz

**Affiliations:** Saint Louis, Mo.; Professor of Obstetrics and Gynecology at Washington University Medical School


### Mechanism and Treatment of Placenta Previa[1]

By HENRY SCHWARZ, M. D., Saint Louis, Mo.
Professor of Obstetrics and Gynecology at Washington University Medical School

TEXTBOOKS and medical journals are at present teeming with conflicting statements regarding maternal and fetal mortality in placenta previa, when treated by the simple means which are at the disposal of every practitioner of medicine, and heroic measures are recommended for the routine treatment of this abnormal condition which, if generally adopted, cannot fail to sacrifice many lives and to bring discredit upon our noble profession.

Therefore, I think it opportune to bring the entire subject of placenta previa up for discussion. We cannot expect to arrive at satisfactory conclusions regarding treatment, unless we make clear to ourselves the mechanism of labor in this pathological condition and the symptoms and dangers which this mechanism involves.

The cause of placenta previa is found in a condition of the uterine mucous membrane, which leads to an implantation of the impregnated ovum in the lower half of the cavity of the womb. Such a condition of the mucosa is apt to follow fre-

---

1, Read before the Alumni Association of the Washington University Medical School, February 25, 1909. From the University Bulletin.

quent child-bearing, for it is a well established fact, that the predisposition to placenta previa increases with the number of labors through which a woman has passed.

Under normal conditions, at the time when the impregnated ovum enters the uterine cavity, the mucosa is found in that state of swelling and softening, which we designate as the decidua menstrualis. The lining is thereby thrown into folds, and the ovum, as a rule, becomes implanted in the upper half of the cavity, changing this decidua menstrualis into the decidua of pregnancy.

When the swelling and the formation of ridges in the upper uterine cavity is imperfect, the ovum is carried down to the lower portion of the cavity, and there it embeds itself by burrowing into the lining and, thereby, stimulating the rapid development of decidua. In this state of swelling there is no hole at the internal os through which the ovum could drop; the anterior and posterior portions of the lining are in close apposition and become firmly blended, when the trophoblast by its corroding and digesting action spreads from one side of the womb to the other.

Hofmeyer's theory, that placenta previa originates from a so-called reflexa-placenta, deals with impossibilities. When an ovum is fully implanted and the chorionic villi cover it on all sides, the system of the allantois being in full operation, those villi, which are embedded in the decidua reflexa, must atrophy from lack of nutrition when the ovum gets larger, while those villi, implanted towards the muscular wall of the uterus in the decidua serotina, are in contact with the maternal bloodvessels, they sprout and prosper and develop into the placenta. It is ridiculous to teach that the opposite takes place, as does the theory of Hofmeyer.

The implantation of the ovum in the vicinity of the internal os, no doubt, often leads to miscarriage during the early month of pregnancy without the true cause of the accident becoming known. At other times there are hemorrhages after the placental circulation is fully established, which may or may not lead to a miscarriage.

The majority of cases, however, which are recognised as cases of placenta previa, cause no symptoms until the second half of pregnancy is reached. At this time the uterine contractions, which occur during the greater part of pregnancy and which increase in frequency and force as pregnancy advances, have attained sufficient force to cause a separation of the lower pole of the ovum from the uterine wall and to cause the internal os to open up so that the examining finger feels the membranes over the presenting part. This opening of the inner os during the latter weeks of pregnancy, is the rule for multiparous women, who furnish the great majority of all cases of placenta previa, but occasionally we also find it to happen during a first pregnancy, so that three or four weeks before labor takes place, we can insert the examining finger through the internal os.

These changes in the lower segment of the womb will of necessity lead to more or less bleeding, if the placenta is so implanted, that a detachment of the lower pole of the ovum must include the detachment of a part of the placenta. In other words, the bleeding at this time depends upon the variety of placenta previa with which we have to deal.

It is customary to speak of three varieties of placenta previa, namely of

*Placenta previa centralis,* or *central placenta previa,* when the center of the placenta is over the internal os.

*Placenta previa partialis* or *partial placenta previa,* when an eccentric portion of the placenta covers the internal os, and of

*Placenta previa lateralis* or *marginalis,* when no part of a low-seated placenta covers the inner os before labor has fairly begun, but when during the progress of dilation a greater or lesser margin of the placenta presents in the dilated os.

The frequency of placenta previa, when thus classified, is perhaps a little greater than the books state.

Williams says in all probability it would be correct to say, that it is met with about once in 1000 cases in private as compared with once in 250 cases in hospital practice.

Statistics are not at all reliable; in hospital practice there is naturally a greater percentage of abnormal cases, because they are transferred to the hospital for the very reason that they are abnormal; and in general practice quite a number of abnormal cases, including placenta previa, remain without record.

One case of placenta previa in every 500 cases of labor seems to me a fair estimate; this would give to the city of Saint Louis with 15,000 births per annum, about 30 cases of placenta previa each year. Of these 30 cases, about one fifth, that is, six in all, would be of the central variety; three fifths, or 18, would be of the partial variety, and the remaining six cases would be of the marginal variety.

The symptoms of placenta previa are hemorrhage and the presence of placental tissue in the dilated os during the first stage of labor.

The hemorrhage is due to the separation of portions of the placenta from the uterine wall; the separation takes place in the decidua serotina, tearing the maternal vessels, which lead into the intervillous spaces of the placenta. This hemorrhage always comes from the wall of the womb and never from the portion of placenta which is detached, and which may fill out the mouth of the womb.

It may show itself early in the second half of pregnancy, and it is bound to return with every further separation of placental tissue from the uterine wall.

The hemorrhage itself, or the treatment which it necessitates, often cause premature labor, so that fully one-half of all children born in cases of placenta previa, are premature; a good many are not viable, while others are only barely so. When labor sets in at term, or prematurely, the mechanism of dilatation is the same as in normal cases.

The contractions of the uterus cause a stretching of the lower segment, that is, the portion of the uterine wall immediately above the inner os and thereby, the lower pole of the ovum becomes separated from the inner os upward to a distance of about ten centimeters; this can be easily verified by the examining finger. The margins of the internal os are next pulled apart, and the bag of waters begins to form and assists in the stretching of the cervix, until dilatation is complete and the membranes are ready to rupture. Up to this time the contractions of the uterus have worked upon the ovum as a whole; with each contraction the whole ovum has been forced downward; its lower end has become detached from the lower segment of the uterus, while the rest of the ovum remains securely adherent to the uterine wall; the fetus has not suffered from the contractions, because the surrounding amniotic fluid has equalised the pressure and the placental circulation has not suffered, because the large bulk of the ovum prevents the uterine wall from contracting to a degree which would cut off the maternal blood-current to the placenta during the labor pain, as is the case in the second stage of labor, when the uterus is so far emptied of its contents by the escape of the amniotic fluid and by the descent of the presenting part into the lower protion of the parturient canal, that during each contraction the uterine wall becomes anemic, and the placental respiration is at a standstill, as shown by the retardation of the fetal heart-beat during the labor-pains.

Now, let us consider, what the same mechanism will do in the various forms of placenta previa. Take first the central variety; the placenta becomes detached for a considerable distance from the inner os upward. The contractions of the womb force the entire ovum downward and compress the bleeding uterine wall, so that there is little or no bleeding during the height of a labor pain, but the moment the uterus relaxes, this compression of the bleeding part ceases and there is, as a matter of course, profuse hemorrhage; toward the end of the first stage the contractions are longer in duration and of greater frequency, and the lower pole of the ovum remains under great tension even during the interval of pains, so that there may be less hemorrhage at that time than there had been at the beginning of the first stage. The hemorrhage, if allowed to go unchecked, may have been fatal before dilatation is complete, but the fact remains, that we often do not reach the patients until the os is fully dilated, or nearly so, and while at times we find the patient nearly exsanguinated, I have never found one that had bled to death unattended, and I have found some that were in very fair condition, although the os was fully dilated.

The fetus up to this time has, as a rule, not suffered at all, for although the central portion of the placenta is detached in a circle of 12 centimeters and more in diameter, the surrounding margin of the placenta still remains attached, and is fully able to carry on a sufficient placental respiration. It is also a fact, that in placenta previa the placenta is usually much thinner and much larger in diameter than when implanted in the upper uterus, so that the portion remaining in function constitutes the greater half of the after-birth.

In cases of partial placenta previa, the hemorrhage is just as severe in the beginning and nothing but placental tissue can be felt in the mouth of the womb, but as dilatation progresses, less placental tissue becomes detached, and when dilation is complete, we find a bag of waters, the walls of which on one side are formed by placental tissue and on the other side by the fetal membranes. In these cases the disturbance of the placental respiration is insignificant.

In the third variety of cases, we find in the beginning of labor only the normal fetal membranes in the mouth of the womb, but as dilatation advances, there is more or less hemorrhage, and a margin of the placenta becomes palpable. The bag of waters consists almost entirely of the fetal membranes.

If the cases were left entirely to nature, and if the child was presenting the vertex, labor would be terminated as follows:

In the partial and the marginal variety of placenta previa the membranes would rupture; the head would descend and would press the detached portion of the placenta against the bleeding uterine surface, stopping all further hemorrhage until the birth of the child, when there would be a return of the hemorrhage because the lower portion of the uterus is slow to contract, and the placenta would most likely be inclined to remain adherent for a considerable time excepting, of course, the part already detached. With the usual assistance however, given to every normal case during the third stage, the placenta comes away promptly; but there remains a greater inclination to post-partum bleeding, requiring a prolonged watching and kneading of the uterus and an early and liberal use of the most potent preparations of ergot.

In the central variety of placenta previa the rupture of the bag of waters, which is formed entirely of placental tissue, cannot take place, until severe pains have detached the entire placenta, when the rupture will occur in the membranes; the placenta will either be born before the head, or it will be pushed to one side and be born immediately after the birth of the child. At any rate, the placenta will be entirely detached before birth can take place; this will mean the death of the child, unless the whole process from the time of complete dilatation, to the birth of the child is completed within a few minutes, as happened in the case reported by Drs. Larew and W. G. Moore of St. Louis, in which labor was so severe and so rapid in succession, that without much bleeding the afterbirth was born and was followed immediately by the birth of a living child.

In most cases such happy results would not be possible, because the hemorrhage is likely to be fatal to the mother and in 25 per cent of the cases the presentation of the child is abnormal, the presence of the bulky placenta in the pelvis preventing the entrance of the fetal head.

Placenta previa, therefore, implies considerable danger to the life of the mother, and still greater danger to the life of the child, and requires prompt, energetic and well directed effort on the part of the obstetrician, just as soon as its existence becomes known.

The danger to the life of the mother may be averted in every case; the danger to the child can also be averted as far as labor itself is concerned, but many children must die, because labor will take place when the child is not sufficiently developed to continue to live.

First assertion. The danger to the mother can be averted in every case.

Second assertion: The child should be delivered in nearly as good a condition as it was in the beginning of labor.

The danger to the mother consists at all times in the hemorrhage. The hemorrhage during the first stage may be fatal; the hemorrhage during the second stage may be fatal; the hemorrhage during the third stage may be fatal, and the hemorrhage post-partum often has been fatal.

But if we can manage to control the hemorrhage until dilatation of the cervical canal is complete, and if at the same time we can preserve the integrity of the ovum, there is no doubt that we ought to be able in either variety to save the mother and to deliver the child alive, if it is living at the time. In the partial and marginal varieties we would rupture the membranes, we would force the head down and deliver quickly, or else, we would turn the child and extract it quickly. In most cases the child is not fully developed and delivery is easy; we would next watch the uterus and express the placenta after Credè's method and, if there should be the least delay, we would detach it manually; in either case, we would give ergot either per mouth or hypodermatically, and we would continue to watch the uterus for several hours.

In the central variety we would, as soon as dilatation is complete, go in with the hand on the side on which the child's feet are; we would detach the placenta from the uterine wall on that side, go up between uterus and membranes, until we were opposite the feet of the child, rupture the membranes, seize the anterior foot, turn the child and deliver at once, taking care of the placenta as in the other cases.

The only question therefore, is: Can we control the hemorrhage during the first stage of labor, and can we preserve the entirety of the ovum during that time?

The answer should be, that we can do this in every case, if we resort to an effective tamponade of the cervix and the vagina.

In every case of pregnancy, whether the woman is in labor or not, such a tamponade becomes necessary, just as soon as she has an alarming hemorrhage.

When we resort to tamponing a pregnant woman, we know that labor is bound to be induced, for to be effective the tamponade must not only fill out the cervix, but it must distend the upper vagina in a way, which is almost sure to induce labor pains.

There are some rare exceptions. On May 19, 1907, a woman who had last menstruated on February 9, 1907, and who was therefore about 14 weeks pregnant, was seized with severe uterine pains and an alarming hemorrhage, requiring tamponade and hypodermic injections of morphine; I removed the patient to St. Luke's Hospital with the intention of cleaning out the uterus. The following day the tamponade was removed; the patient had no more pains and the bleeding had entirely ceased, the mouth of the womb remained closed. After two weeks, the patient returned home; she had no more bleeding until September 9, when a slight hemorrhage required a few days of absolute rest. November 26, labor set in with moderate hemorrhage; the case proved to be one of partial placenta previa and the patient, a primipara, was delivered of a living child.

In all other cases that have come under my observation, whenever an effective tamponade had been resorted to, labor pains would invariably follow, if they were not present at the time.

It is claimed by those who recommend the heroic treatment for placenta previa, that the tamponade cannot be made effective and that it tends to septic infection, and therefore, they recommend and practice the version after Braxton Hicks, which consists in turning the child just as soon as there is enough dilatation for one or two fingers, after perforating the ovum, usually through the placenta, using the child's tapering body to plug the lower segment of the uterus and leaving the case to nature. The integrity of the ovum is hereby destroyed; the chance for infection is increased, the child's life is sacrificed and the normal detachment of the placenta is interfered with. I have never performed version after Braxton Hicks, and have always advised against it; yet it has been the universal custom the world over during the last twenty-five years, partly because the leading German obstetricians followed Hofmeyer, who has been one of the champions of this pernicious practice.

Of late, attempts are being made to save a greater number of children, by endangering a greater number of mothers, and Cesarean section is being recommended as the routine treatment of placenta previa.

About twenty years ago, Dr. Ford of St. Louis, recommended Cesarean section in cases of placenta previa and one of our local surgeons, Dr. Bernays, soon put the advice into practice, (*Jour. Amer. Med. Assoc.*, 1894), but luckily he did not find many followers.

With the advent of Bossi's dilator, an instrument which is a great addition to obstetrical resources, much damage has been done by using it to force a dilatation in cases of placenta previa, for which cases it is entirely unfit, because in these cases dilatation should never be forced, the tissues being especially prone to lacerations on account of the implantation of the placenta in the lower segment of the uterus; by this unadvised use of the steel dilator discredit has been brought upon a deserving instrument.

Vaginal Cesarean section has likewise been advised and practised in cases of placenta previa, and has already killed a good many women, whose lives should have been saved.

If a properly executed tamponade controls the hemorrhage in placenta previa until dilatation is complete, and if, at the same time, it preserves the entirety of the ovum, there can be no room nor justification for the employment of any means which interfere with nature's mechanism, such as forcing dilatation with dilators, perforating the placenta and turning the child after Braxton Hicks, perforating the placenta and introducing rubber-bags, and least of all vaginal and abdominal Cesarean section. The latter operation appears criminal when we consider that no sane man would advise it after there is complete dilatation of the cervix, and that the most experienced obstetrician can not tell whether the case is one of central or of partial placenta previa until dilatation is complete.

Unfortunately, some of the foremost teachers in Germany have of late assumed a rather aggressive position regarding the treatment of placenta previa, and there is great danger that their teachings will have an injurious effect on the practice of midwifery in our country; not enough, that all of them consider the version after Braxton Hicks as the proper treatment, Professor Henkel of Griefswald, (*Archiv für Gynecologie,* Vol. 86), advocates vaginal Cesarean section in every case, and Professor Kroenig, of Freiburg, (*Centralblatt für Gynecologie,* 1908, No. 46), declares abdominal Cesarean section to be the ideal treatment for all cases of placenta previa, if one can get hold of them early enough, that is to say, before the progress of labor has singled out the 20 per cent. of central placenta previa from the 80 per cent. of partial and marginal placenta previa and before dilatation is complete, at which time even he has not the hardihood to recommend Cesarean section.

Yet he states, that during the last year he has performed Cesarean section for placenta previa six times; all six women left the bed during the first twenty-four hours after delivery; all six women left the clinic during the first three weeks; all six children were born alive; only one died before the mother left the hospital; in no case was there any post-partum hemorrhage requiring the packing of the uterus. Kroenig has likewise performed a number of vaginal Cesarean sections for placenta previa; a few of them did nicely, because the placenta was attached to the posterior wall of the uterus, but when he struck two cases in which the placenta was attached to the anterior wall of the uterus, the hemorrhage was so great that both patients died.

The truth is, that if the placenta was attached either to the anterior or to the posterior wall of the uterus, he dealt with cases of partial placenta previa, and by a more rational mode of treatment, the lives of these mothers would have been saved. If the mouth of the womb is nearly or fully dilated when the cases reach Professor Kroenig, he performs version and leaves the case to nature; that is, he sacrifices the child if the cases reach him after they have been tamponed, he performs vaginal Cesarean section, if the placenta is attached posteriorly; but if it is attached anteriorly, he turns the child after Braxton Hicks, sacrificing it and exposing the mother to the added dangers of protracted labor, because he imagines that none of the general practitioners in his district could use the tamponade without infecting the patient.

Professor Kroenig takes great unction upon himself, because his six cases of placenta previa did not have sufficient post-partum hemorrhage to require packing of the womb. I think it is about time that this talk of checking post-partum hemorrhage by packing the uterus be stopped. Whenever packing the uterus with gauze or any other material stops post-partum hemorrhage, the packing was not necessary, because the packing can only have effect, if the uterine wall is well contracted, so that it has a hard surface to press against; and of course, if the womb is well contracted, there can be no hemorrhage. On the other hand, it is an old established maxim, that if you wish the uterus to contract well, you must remove placenta, blood-clots or other contents, which interfere with efficient contractions and therefore, packing the uterus is contrary to all reason.

It now remains for me to prove that it is possible to control the hemorrhage in all cases of placenta previa until dilatation is complete by tamponing cervix and vagina, without either infecting the case or destroying the entirety of the ovum.

In 1883, Dr. Jungbluth, of Aix-la-Chapelle, published (*Volkmann's Vorträge* No. 235), a method of tamponing by means of specially prepared sponge-tents and reported seven cases of placenta previa so treated, in which every woman was saved and every child was delivered in the condition it was in when labor began. I prepared at that time some sponge-tents after Jungbluth's method, but I have never used them, because I found that packing with cotton, squeezed out in 4 per cent. solution of carbolic acid and rammed tight with the aid of speculum and dressing forceps, answered every purpose. Of late years, I have preferred to use sterile gauze bandages for packing, but either material will do; the only precaution necessary is to pack tightly enough, and to remove the tampons every 6 or 8 hours, unless severe labor pains force you to remove them sooner. In handling a fair number of cases of placenta previa of all varieties, I have not in one single case lost a mother or had infection follow the use of the tamponade.

The number of cases is far above fifty, counting all varieties; they have all happened in the practice of medical friends, who can easily repudiate my statements, if I should exaggerate. I mention in detail only a few cases, which show that tamponing cervix and vagina until full dilatation has taken place, was possible and safe, twenty and more years ago, when things were not as perfect as they are now and, of course, the method is much safer now than it was then. I also wish to illustrate the way in which the practitioner sees most of his cases, and which makes a transfer to a hospital often unnecessary and usually impossible.

Case I. July 24, 1881, I was called to Mrs. L., in Neuenheim, across the Neckar from Heidelberg; found the patient highly anemic, swimming in blood, and a pool of blood under her bed. A quick examination showed the os dilated to about 8 centimeters; the bag of waters was very tense, ready to burst; it consisted on the right half of placental tissue, on the left half of fetal membranes; there was much forewater; both feet with the heels toward the right side could be felt through the membranes; I introduced the full hand, ruptured the membranes and extracted the child, without delay. The placenta followed easily after Crédè's method and there was no further hemorrhage. The uterus was watched for several hours; full doses of ergot were given three times a day for two days. The patient made an uneventful recovery and the child, a boy, remained alive.

The history taken later showed that this was the fourth pregnancy, which had advanced to the thirty-first week. Four weeks previous, there had been a severe hemorrhage, which had stopped by itself; during the last two weeks, there was slight bleeding every two or three days. The case was in charge of a midwife; pains had started the previous day at 4 P. M. On July 24, at 5.30 A. M., the pains became severe and there was at the same time a severe hemorrhage; much time was lost in sending first for two other physicians, who could not take charge of the case, so that I did not get to the patient until over four hours after the bleeding had started. This case illustrates, that in cases of partial placenta previa, if left to nature, complete dilatation may take place without serious injury to mother and child.

Case II. Mrs. K., Grossmandelgasse, Heidelberg, has had three normal confinements. On October 27, 1881, at 2.30 A. M., I was called, found the patient highly anemic and swimming in blood. The vagina was full of clotted blood, which I removed; the mouth of the womb was as large as a mark (about 2 cm), and nothing but placental tissue was palpable; I gave a hot douche and tamponed tightly; the bleeding stopped. The presenting part was not palpable from the vagina; fetal heart-sounds were normal in the right lower quadrant of the abdomen; the head was freely movable above the pelvic inlet. The history showed that the patient menstruated the last time early in May, and that she was therefore, less than seven months pregnant; pregnancy had been normal up to October 26, when there was a moderate hemorrhage and the family physician advised rest in bed; at 1.30 A. M., October 27, the woman awoke, finding herself swimming in blood; she called her husband and fainted away. After the tamponing, I lowered the patient's head and advised the free drinking of milk and water. When I returned at 8 A. M., there had been no bleeding; labor pains were regular, but not very strong; the head had escaped to the left iliac fossa; the breech could be felt to the right above the navel; the fetal heartbeat was 144.

The patient was transferred to the Lying-in Hospital; here the tampons were removed; the os was found nearly dilated, the left half was filled out with placental tissue, in the right half the membranes were palpable. Professor Kehrer ruptured the membranes and turned and extracted the child, which was premature, showing less than 26 weeks development and died after 10 minutes. The placenta followed easily; the puerperium was uneventful. This case illustrates the fact that after a severe hemorrhage, in the middle of the night and in poor surroundings it is possible to employ effective tamponade, from the beginning of labor until dilatation is complete, without infecting the patient

Case III. December 7, 1888, at 11 P. M., I was called to a 20 year old primipara whom Dr. Bierwirth had tamponed on account of severe hemorrhage. I removed the tampon and gave a vaginal douche; the mouth of the womb barely permitted the passage of one finger; the tamponade was renewed; fetal heart-sounds could not be heard; head movable above inlet; back to the left; at 8 A. M. the patient was chloroformed; the tampons were removed; the os was found nearly dilated and entirely filled out with placental tissue. The left hand was introduced; the placenta peeled off from the right side of the uterus, the hand forced up between membranes and uterine wall until the right knee was reached; the membranes were now ruptured; the child turned and extracted; the rest of the placenta was now peeled off and placenta and membranes removed without difficulty; the uterus was watched for some time and fluid extract of ergot was given in repeated doses. The puerperium was uneventful. The child was still-born and showed beginning maceration.

Case IV. July 10, 1889, at 8 P. M., I was called by Dr. Gerling to a case of placenta previa giving the following history: The patient is 34 years old and in the eighth month of her fourth pregnancy. In the afternoon, while visiting a neighbor, she is taken with a severe flooding spell; she is carried home and Dr. Gerling is sent for, who tampons the vagina, thereby stopping the hemorrhage. There was no bleeding when I arrived and I therefore did not renew the tampons until 11 P. M., six hours after they had been introduced. The child presented the vertex with the back to the left; the fetal heart-beat was normal. On renewing the tampons we found the mouth of the womb barely permitting the passage of two fingers and filled out entirely with placental tissue; toward morning severe labor pains set in and at 7 A. M., the patient is chloroformed; the tampons are removed; the os is found fully dilated and filled out entirely with placental tissue; the left hand is introduced; the placenta peeled off on the right side; the membranes are ruptured, the child is turned and extracted. The placenta comes away with ease; the puerperium was uneventful; the child was about eight weeks premature but remained alive.

These two cases illustrate the fact that general practitioners twenty years ago were well able to pack the vagina effectively and aseptically in cases of central placenta previa.

CASE V. August 27, 1889, at 7 P. M., I was called by Dr. Wichmann to go with him to a case of severe flooding during labor. Mrs. D., 31 years old, was in labor at the end of her eighth pregnancy, when the hemorrhage occurred. On our arrival, we found the woman weak from loss of blood; the vagina filled with clotted blood; the os fully dilated and filled out entirely with placental tissue; I peeled the placenta off on the right side, ruptured the membranes and turned the child; as soon as the hemorrhage stopped, on account of the child's breech plugging the bleeding uterine vessels, I paused long enough to have the patient put under chloroform, when I extracted a living male child of normal dimensions. The placenta followed with ease and the puerperium was uneventful.

This record illustrates that cases of central placenta previa may go to full term and may go unassisted to complete dilatation, without serious damage to mother or child.

It is not necessary to weary you with reports of many cases. The ones reported, I picked for a purpose. The two cases of the year 1881 and 1882, which date from the time, when I was chief assistant to Professor Kehrer at Heidelberg, and when I was building up the first obstetrical outclinic at that German university, show, that at that time and at that place, students were taught to control the hemorrhage in placenta previa with the aid of the tampon until full dilatation had been attained, when both mother and child could be saved by the classical version. The transfer of the second case to the Lying-in Hospital, was mainly done for the purpose of having the patient delivered before a class of students; we not only made the students practice versions on the manikin, but we made them practice the tamponade on clinic cases; the tamponade was also taught to the future midwives, of whom I handled a class of over sixty every year; for them we secured vagina and uterus of female calves and suspended them in a manikin, which furnished very satisfactory practice.

The other cases were picked to show the efficiency of the tamponade years ago, before things were quite as convenient as they are now, and likewise to show that there was no reason in the world, why this beneficial practice should have been changed at any time.

At present we proceed as follows: Any case of moderate bleeding during pregnancy is put to bed and a competent nurse is placed on watch with the material ready to pack cervix and vagina tightly in case of necessity; in all severer forms of bleeding during pregnancy or during labor, we give a hot douche and pack cervix and upper vagina very firmly with large balls of absorbent cotton squeezed out in carbolated water, so that the cotton is no longer elastic and will no longer absorb. (A 4 per cent. carbolic acid solution is used in preference to a solution of mercury bichloride, because tampons saturated with the latter solution become offensive in a much shorter time than do those saturated with the carbolated solution, presumably because part of the bichloride combines with the albumen of the blood and becomes inactive.)

The cervix is exposed in a speculum (either Sims or the anterior and posterior retractors); seized with strong vulcellum-forceps; pulled down and packed so as to leave no space for blood to collect. In so packing, there is no danger of detaching a greater area of placenta, but should this happen, it would only be an advantage, as it would save nature considerable time; in fact, the old advice of Barnes, to introduce a finger through the internal os and to sweep it around in a circle, detaching the placenta for a considerable distance, is a very good one. Nature must first detach the placenta for just such a distance before dilatation is possible, but there is no bleeding between the uterine wall and the placenta, because the uterine contractions press the entire ovum, with its detached lower pole, tightly against the bleeding surface. After packing the cervix, the upper vagina is packed so tightly, that it presses firmly against the lower portion of the uterus and likewise, holds the bleeding surface against the lower pole of the ovum, and in this manner the hemorrhage is easily controlled. The lower vagina is packed firmly with dry cotton or with dry gauze, and the whole packing is held in position by a T-shaped bandage. We now use sterile rubber-gloves during these manipulations and the specula and dressing forceps used for the packing, are sterilised by boiling; twenty-five years ago they were simply placed in antiseptic solutions.

I have, since 1889, seen a fair number of the various forms of placenta previa, and have asked a number of my medical friends, in whose practice these cases have occurred, to be present here tonight, to corroborate my statement when I say, that of these fifty and more cases, I have never lost one mother and that the children were born in about the same condition they had been in, when the case came under observation.

There should be one exception to this statement in so far as in one case, which I reported in the American Text-Book of Obstetrics, a doctor friend of mine not only tamponed effectively in a case of central placenta previa, but likewise gave a teaspoonful of ergotol. This produced terrific contractions; the packing and the placenta filled out the pelvis so completely, that the child could not enter and the uterus ruptured before I arrived on the scene. I removed the packing and delivered the woman; the child had almost completely escaped into the abdomen; the woman died soon after delivery.

We should remember, that cases of placenta previa must of necessity be handled by the general practitioner more than by the expert in well equipped clinics, and that for this reason, it is the simple duty of the teacher to handle his cases in such a way, that his pupils can go out and follow his example.

A qualified obstetrician needs neither instruments nor apparatus of any kind to deal with any form of placenta previa in case of emergency. Suppose while out hunting, you find yourself the only visitor on an out-of-the-way farm: and suppose, during your visit the farmer's wife, who is seven or eight months pregnant, is seized with a frightful uterine hemorrhage; would you let her die, because you have only your hand and your head to support you in this emergency? Certainly not; you would place the woman with her head low; you would wash your hands as thoroughly as circumstances permit, you would cause a kettle with water to be put over the fire, and you would tear bed-sheets, or any other material, no matter how little aseptic it may look, into strips and boil them, knowing that thereby they are rendered absolutely aseptic. You would remove the clots from the vagina and pack the vagina tightly until dilatation is complete. In the mean-time you would have more time than enough to make reasonable preparations for delivery. You would finally remove your packing, detach the placenta, turn and extract the child; remove the placenta; hold your hand over the uterus as long as necessary, and the chances are you would save both mother and child.

What we need in this country is better obstetrical training for the general practitioner, and Germany needs this even more than we do, in spite of the splendid records of its University Clinics and the individual work of its leading teachers. Professor Kroenig mistrusts the ability of the doctors and midwives in the district from which he draws his cases, to effectively and aseptically pack the vagina and to perform version in cases of placenta previa, and therefore he wants them to send these cases to his clinic at Freiburg, at the risk of the patient's life, instead of teaching them these things properly, as is his plain duty.

In conclusion let me relate the saddest case of placent previa, which has ever come to my notice, and which happened in Professor Kroenig's district in the winter of 1881 to 1882.

The Feldbergerhof is the hostelry near the summit of the Feldberg, the highest mountain of the Black-Forest; the nearest settlement is Lenzkirch, on the eastern slope of the mountain. In September, 1882, I spent the better half of a day in that little inn, waiting for a severe rainstorm to subside, so that I might go to the summit.

The keeper, whose name was Meyer, learning that I was attached to the Lying-in Hospital at Heidelberg, told me his story. They had several small children; his wife was pregnant when winter came on, and communication with the outside world was cut off; the event was not expected until in spring when it would be possible to get a midwife or a doctor from the valley below. But in the middle of winter, when it would have been suicidal to attempt a descent into the valley, his wife was seized with hemorrhage; he used what home remedies he possessed, without any effect; she bled and she bled for nearly twenty-four hours, and finally died in his arms, undelivered. Thus he and his little children, cut off from the world, fierce snowstorms raging around their hut, were left alone with the dead wife and mother, who had died from placenta previa.

440 N. Newstead Avenue.

